# Knowledge of newborn healthcare among pregnant women: basis for promotional and educational programs on breastfeeding

**DOI:** 10.1590/S1516-31802001000100003

**Published:** 2001-01-02

**Authors:** Hugo Issler, Márcia Borges Sanches Rodrigues de Sá, Dulce Maria Senna

**Keywords:** Breast-feeding, Promotional programs, Educational programs, Aleitamento materno, Promoção do aleitamento materno, Programas educativos

## Abstract

**CONTEXT:**

Promotional and educational programs relating to breastfeeding are important for reversing the decline in this practice. Most programs are centered exclusively on breastfeeding, although general knowledge about newborn healthcare may be important, especially among pregnant women.

**OBJECTIVE:**

To study pregnant women's knowledge about general healthcare of newborns, including breastfeeding aspects.

**TYPE OF STUDY:**

Cross-sectional.

**SETTING:**

Prof. Samuel Barnsley Pessoa Health School Center, Faculty of Medicine, Universidade de São Paulo, Brazil.

**PARTICIPANTS:**

All pregnant women who were registered in the prenatal care program during six consecutive months.

**MAIN MEASUREMENTS:**

Aspects of the current gestation, previous gestations and childbirths, knowledge of the general aspects of newborn healthcare and of breastfeeding practices.

**RESULTS:**

The results show that only a little over half of the pregnant women had received any information on newborn healthcare. Misinformation was clearly present regarding proper care of the umbilical stump and the nature of jaundice, and worst regarding how to treat oral thrush and jaundice, and about vaccination. In relation to breastfeeding, even though almost all the pregnant women declared their intention to breastfeed, less than half had a concrete response regarding how long to do it for. The low rates obtained in the topics dealing with the duration, nursing intervals and the attitude to be taken towards hypogalactia show unfamiliarity with the breastfeeding technique. The “weak milk” belief, the misinformation about contraceptive methods during breastfeeding and the cost of artificial formulas also have a negative impact on this practice.

**CONCLUSIONS:**

Pregnant women's knowledge of newborn healthcare is low, as much in the aspects of general care as in relation to the practice of breastfeeding. These findings must be taken into consideration in educative programs promoting breastfeeding.

## RESUMO

**CONTEXTO:**

Os programas de incentivo ao aleitamento materno são importantes para reverter o declínio desta prática. Muitos desses programas têm se fixado em discutir exclusivamente aspectos do aleitamento materno, quando conhecimentos gerais sobre cuidados de saúde para recém-nascidos podem ser demanda importante, especialmente entre gestantes.

**OBJETIVO:**

Estudar conhecimentos de gestantes sobre cuidados gerais de saúde de recém-nascidos, incluindo aspectos referentes ao aleitamento materno.

**TIPO DE ESTUDO:**

Inquérito transversal.

**LOCAL:**

Centro de Saúde-Escola “Prof. Samuel Barnsley Pessoa” da Faculdade de Medicina, Universidade de São Paulo.

**AMOSTRA:**

Todas as gestantes inscritas no programa de cuidados pré-natais durante seis meses seguidos.

**VARIÁVEIS AVALIADAS:**

Aspectos da gestação atual, eventuais gestações e partos anteriores, conhecimentos sobre aspectos gerais da saúde de recém-nascidos e sobre a prática do aleitamento materno.

**RESULTADOS:**

Os resultados mostram que apenas pouco mais da metade das gestantes recebeu alguma informação sobre cuidados de saúde para recém-nascidos. É clara a desinformação quanto ao tratamento adequado do coto umbilical, à noção do que seja a icterícia neonatal e, ainda mais grave, em relação ao tratamento da monilíase, da icterícia e vacinação. Em relação ao aleitamento materno, embora quase todas as gestantes declaram intenção de amamentar, menos da metade tinha proposta concreta quanto a duração da amamentação. Os baixos índices obtidos nos tópicos que tratam da duração, intervalos de mamada e sobre a atitude a ser tomada na hipogalactia mostram desconhecimento da técnica de aleitamento. A crença do leite fraco, a desinformação de métodos contraceptivos durante a amamentação, do custo da alimentação artificial formam um contexto também negativo para esta prática.

**CONCLUSÕES:**

Os conhecimentos das gestantes entrevistadas sobre cuidados de saúde para recémnascidos são precários, tanto no aspecto de cuidados gerais, como também em relação à prática do aleitamento materno. Estes achados devem ser levados em consideração nos programas educativos de incentivo ao aleitamento materno.

## INTRODUCTION

Today, the importance of breastfeeding is widely recognized.^1-6^ However, early weaning remains a challenge in developing countries.^7-11^ This can be attributed to the process of urbanization and industrialization,^12^ although the reasons are complex and varied.

In urban areas the extended family, i.e. grandparents, uncles and cousins, has been replaced by the nuclear family. As a result, there has been a break in the knowledge transmission chain from the older to the younger women in a family. When we observe the breastfeeding process in more traditional societies, we may be deceived by the apparent simplicity of the process. It is an error to conclude that all women are prepared for lactation. In fact, the art of breastfeeding is not innate, but one that has been taught from one generation to the next since time immemorial.^13^ A girl that lacks the company of family women other than her mother has few opportunities to watch and learn mother-to-child care techniques. When she becomes a mother herself and returns home after delivery, there are usually no close relatives she can count on, thereby finding herself isolated and unprepared. The nuclear family structure is one of the main reasons for the decline in breastfeeding in urban areas.^14^

However, breastfeeding is only part of the necessary newborn care techniques. These include childcare procedures that create the context needed for normal growth and development. Once the obstacles to the transmission of knowledge are identified, alternatives like breastfeeding promotion programs are needed; especially those aimed at pregnant women.^15^

Therefore, a broad survey on the knowledge of newborn healthcare among pregnant women was devised, aimed both at general health care topics and breastfeeding. Its results may prove useful in establishing new promotional and educational programs in relation to breastfeeding.

## METHODS

### Setting

The “Centro de Saúde-Escola Prof. Samuel Barnsley Pessoa” *(Prof. Samuel Barnsley Pessoa Health School Center)* is a primary health care facility located in a poor area of São Paulo that has been created as a joint effort between the University of São Paulo and the São Paulo State Health Office. It serves a population of 50,000 adults and children, and offers a prenatal care program. The Pediatrics and Preventive Medicine Departments of the University of São Paulo School of Medicine develop academic and welfare programs there.

### Participants

The sample consisted of 65 pregnant women enrolled for six consecutive months in the prenatal care program of the Prof. Samuel Barnsley Pessoa Health School Center. The population consists mainly of low-income migrants, living in shanties or slum tenement houses. The overall literacy rate is low. Most of the family providers do manual labor as construction workers or performing menial tasks.

The study sample was made up from all the pregnant woman enrolled during six consecutive months in the pre-natal care program, a total of 67 subjects. At the end of the program there were 65, as two subjects were excluded due to intended abortion.

The research plan was presented to each pregnant woman at the first prenatal consultation and her collaboration was requested. She was informed that a refusal would not prejudice her clinical care. All the women agreed to participate and were individually interviewed by trained healthcare officers.

The following topics were included in the questionnaire: a) Current prenatal information; b) Prenatal history and parturition; c) Basic knowledge of newborn healthcare; d) Basic breastfeeding practice.

## RESULTS

### Current gestational data

Of 65 subjects, 36 (55.4%) were at up to 14 weeks of pregnancy, 19 (29.2%) from 14 to 28 weeks, 4 (6.1%) over 28 weeks and in 6 cases (9.2%) the gestational age could not be determined; 21 (32.3%) were primigravidae. Of the 44 subjects with a previous pregnancy, 5 (11.4%) had had a previous abortion and 2 (4.6%) a stillbirth. None had produced twins.

### Knowledge sources

The surveyed topics relating to the knowledge source are shown in Table 1. Analysis of Table 1 shows that only 38 (58.5%) of the subjects acknowledged having previous information. Of those 38, 12 (18.5%) acknowledged having had formal instruction from school, lectures or the healthcare center. The remainder was from informal sources, such as family or friends.

**Table 1:** Knowledge Sources on Newborn Healthcare and lactation

Topics	%	*
Previous knowledge	58.5	(38/65)
Formal knowledge	18.5	(12/65)
Experience prior to adolescence	67.7	(44/65)
Watching her own mother breastfeeding	44.6	(29/65)
Knowing she was breastfed	78.5	(51/65)
General information on newborn healthcare given upon discharge from the maternity hospital	58.1	(25/43)
Instruction for breastfeeding given upon discharge from the maternity hospital	39.5	(17/43)

* *number of subjects with an affirmative response and the respective total.*

With regard to previous experience, 44 (67.7%) had helped baby-sitting a child, 29 (44.6%) watched their own mothers breastfeeding their siblings, and 51 (78.5%) acknowledged being breast-fed as an infant.

The 43 subjects who had had at least one live child were asked about the kind of information given upon discharge from the maternity hospital. Only 25 (58.1%) acknowledged having been informed of how to take care of a child, and 17 (39.5%) how to breastfeed.

### General newborn healthcare

Less than half of the subjects knew how to take proper care of the umbilical stump, deal with oral thrush care, or had knowledge of the nature of jaundice. Only 7 (10.7%) answered correctly how to treat jaundice, and 19 (29.3%) when to start vacci-nation. None of the subjects knew what the first 3 vaccines their child should receive were (BCG, Sabin and DPT, on the state of São Paulo schedule). Finally, 47 subjects (72.3%) acknowledged the need for in-house support from family or friends upon arrival home from the maternity hospital (Table 2).

**Table 2:** General knowledge of newborn healthcare

Topics	%	*
Umbilical stump care	46.2	(30/65)
Information about the nature of jaundice	49.2	(32/65)
Treatment of jaundice	10.7	(7/65)
Oral thrush care	15.4	(10/65)
When to start vaccination	29.3	(19/65)
First vaccines	zero	(zero/65)
Need for in-house support	73.2	(47/65)

* *number of subjects with a correct or affirmative response and the respective total.*

### Breastfeeding practice

Regarding knowledge of breastfeeding practice, all the subjects recognized the importance of breastfeeding for the child's health (Table 3). In spite of their assertion, 64 (98.5%) intended to breast-feed, but only 31 (47.7%) were able to give a time frame for weaning.

**Table 3:** Knowledge of breastfeeding practices

Topics	%	*
Importance of breastfeeding for the child's health	100	(65/65)
Intention to breastfeed	98.5	(64/65)
Suggestion for weaning time	47.7	(31/65)
Need for breast care	89.2	(58/65)
Self demand	56.9	(37/65)
Non-timed lactation	64.6	(42/65)
Proper attitude towards hypogalactia	29.2	(19/65)
Overnight nursing	95.4	(62/65)
“Weak milk” belief	46.2	(30/65)
Contraception during lactation	41.5	(27/65)
Nature of artificial formulas	10.8	(7/65)
Cost of artificial formulas	56.9	(37/65)

* *number of subjects with a correct or affirmative response and the respective total.*

Even when lacking knowledge of proper techniques, 58 subjects (89.2%) stated the need for breast care. Regarding the timing of nursing, 37 (56.9%) wanted to nurse on demand, 42 (64.6%) preferred non-timed lactation and 62 (95.4%) acknowledged the need for overnight nursing.

When questioned about hypogalactia, only 19 (29.2%) stated that the mother should increase the feeding frequency, not completing with a bottle. Thirty subjects (46.2%) recounted the belief about “weak milk”. On the topic of contraception, 27 (41.5%) had proper knowledge on how to avoid a new pregnancy during lactation.

Regarding the human milk substitutes, only 7 subjects (10.8%) knew that artificial formulas were made from cow or soybean milk. And 37 (56.9%) acknowledged the impact of the cost of the artificial formulas on the household income.

## DISCUSSION

It is a matter for concern that 58.5% of the women surveyed had limited breastfeeding knowledge. The subjects with previous childbearing experience were poorly informed about the basic topics in newborn healthcare and breastfeeding upon discharge from the maternity hospital. This pattern of deficiency and its need for correction have not only been described in women of reproductive age,^16-19^ but also among obstetricians and pediatricians,^20^ who are at present of great importance in the knowledge transmission chain.

Among the women surveyed, their sources of knowledge were mostly informal, from family or friends. Misinformation was clearly present, for example regarding proper care of the umbilical stump and the nature of the jaundice among newborns. It was worst in how to treat oral thrush and jaundice. The complete lack of awareness regarding vaccination was astonishing, considering the public advertisement it is given.

Recognition of the need for family support is parallel to the knowledge deficiency seen above. Ideally, when returning home from hospital women would have full family support, with help from their mothers and older women. However, the nuclear family structure in urban areas does not offer these favorable conditions, and therefore, institutional help is crucial.

As for the practice of breastfeeding, although its benefits are widely recognized and almost all subjects declared their intention to breast-feed, their knowledge was precarious. The low scores obtained in the topics concerning the timing of nursing and the attitude towards hypogalactia provide evidence of the women's lack of technique, and this will lead to difficulties in sustaining breast-feeding.

The other aspects raised may also lead to early weaning. The belief of the “weak milk” myth, the ignorance about contraceptive methods during lactation and about the cost of artificial formulas may be due to lack of explanation. On the other hand, the misinformation about the nature of artificial formulas may be related to an idea of modernity and sophistication. This idea may explain how readily mothers substitute human milk with artificial formula.

Reviewing the published literature on programs promoting breastfeeding among pregnant women, there is evidence of an increase in duration^21-23^ and of an increased incidence of breastfeeding,^24,25^ but other authors describe the inefficiency of these programs.^26,27^

In view of the contradictory theories, one hypothesis can be made. As a number of authors state,^28-30^ a decision in favor of breast-feeding mostly occurs before pregnancy, and is consolidated during the first trimester of pregnancy. Therefore, it is made before contact with health professionals, and thus a change of attitude would be rare and unexpected. Another reason could be the lack of quality and administrative support for these programs.^31^

The quality of knowledge and support has a crucial role in the success of breastfeeding promotion.^15^ It is worth mentioning that the focus of most of these programs is solely on breastfeeding, without discussion of other topics. Valdés et al.^32^ point to this fact and emphasize the need for inclusion of other infant health aspects in the breastfeeding programs.

From the information above, the need for breastfeeding promotion is clear, and though they may not influence the decision, higher quality programs will increase acceptance and the chance of better results. These survey results are consistent, since there is a relationship between newborn healthcare in general and the practice of breastfeeding, to ensure the care needed for the newborn's survival. Therefore, the acknowledgement of other healthcare topics can enhance promotional and educational programs that relate to breastfeeding.

## CONCLUSIONS

Knowledge of basic newborn healthcare practices among pregnant women is low. Its source is mostly informal, denoting the lack of efficiency of the educational programs.

Knowledge of breastfeeding practices is inadequate, leading to difficulties in relation to lactation success.

Knowledge of newborn healthcare among pregnant women should be considered during the creation of breastfeeding promotion and educational programs.
